# Metabolic Syndrome With Concomitant Anaemia Among Patients With Type 2 Diabetes Mellitus in the Volta Region of Ghana: A Cross‐Sectional Study

**DOI:** 10.1155/jnme/1310197

**Published:** 2026-04-13

**Authors:** Sylvester Yao Lokpo, David Avoyi, Eric Boateng, Samuel Ametepe, Francis Abeku Ussher, Michael Appiah, Precious Kwablah Kwadzokpui, Esther Ngozi Adejumo, Christian Obirikorang, James Osei-Yeboah, Kenneth Ablordey, Abigail Ibrahim

**Affiliations:** ^1^ Department of Medical Laboratory Sciences, School of Allied Health Sciences, University of Health and Allied Sciences, Ho, Ghana, uhas.edu.gh; ^2^ Department of Medical Laboratory Science, Faculty of Health and Allied Sciences, Koforidua Technical University, Koforidua, Ghana, ktu.edu.gh; ^3^ Department of Medical Laboratory Sciences, Accra Technical University, Greater Accra Region, Accra, Ghana, atu.edu.gh; ^4^ Laboratory Department, Ho Teaching Hospital, Ministry of Health, Ho, Ghana, moh.gov.gh; ^5^ Department of Medical Laboratory Science, School of Public and Allied Health, Babcock University, Ilishan-Remo, Ogun State, Nigeria, babcock.edu.ng; ^6^ Department of Molecular Medicine, School of Medicine and Dentistry, Kwame Nkrumah University of Science and Technology, Kumasi, Ghana, knust.edu.gh; ^7^ School of Public Health, Kwame Nkrumah University of Science and Technology, Kumasi, Ghana, knust.edu.gh; ^8^ Department of Adult Health, Nursing and Midwifery Training College, Ministry of Health, Odumase-Krobo, Accra, Ghana, moh.gov.gh

**Keywords:** anaemia, comorbidity, metabolic syndrome, Type 2 diabetes mellitus

## Abstract

**Introduction:**

Anaemia and metabolic syndrome (MetS) worsen the pathogenesis of Type 2 diabetes mellitus (T2DM), leading to increased diabetes‐related complications. This study aimed to assess the prevalence and associated factors of anaemia–MetS comorbidity in individuals with T2DM in the Ho municipality.

**Methods:**

A cross‐sectional study was conducted between January and October 2022 at the Ho Municipal Hospital, targeting patients aged ≥ 20 years with T2DM who were conveniently selected from the diabetic clinic. Data on sociodemographic and clinical characteristics were collected using semistructured questionnaires. Anthropometric and blood pressure measurements were performed. Fasting blood samples were obtained to measure glucose, lipid profile and haemoglobin (Hb) levels. MetS was defined using the harmonized criteria, and anaemia was defined according to WHO gender‐specific cut‐offs. Logistic regression models were utilized to determine factors associated with anaemia alone and anaemia–MetS comorbidity.

**Results:**

A total of 193 participants were enrolled (61.7% female). The prevalence of MetS was 64.8% (*n* = 125), while anaemia prevalence was 23.8% (*n* = 46). Anaemia–MetS comorbidity was observed in 33 participants (17.1%). Anaemia with elevated fasting blood glucose (FBG) was the most frequent combination (23.8%). In the multivariable analysis, female sex was significantly associated with higher odds of anaemia–MetS comorbidity in the sociodemographic model (OR = 3.51; 95% CI: 1.34–10.31), but this association was attenuated after full adjustment. Current alcohol consumption was independently associated with substantially reduced odds of anaemia–MetS comorbidity in the fully adjusted models (OR = 0.10; 95% CI: 0.01–0.54).

**Conclusion:**

Anaemia–MetS comorbidity was common among patients with T2DM. Alcohol consumption was associated with lower odds of comorbidity, suggesting a potential protective effect that warrants further investigation. Female sex may confer increased risk, though this relationship is confounded by lifestyle and clinical factors.

## 1. Introduction

Diabetes mellitus (DM) is a group of metabolic conditions with different causes, marked by consistently elevated blood glucose levels that lead to disruptions in the metabolism of carbohydrates, fats and proteins [1]. Recent data from the World Health Organization (WHO) show that the increase in DM cases is occurring more rapidly in low‐ and middle‐income countries, with worldwide figures growing from 108 million in 1980 to 422 million in 2014 [1]. In 2018, DM directly resulted in 1.5 million deaths, with 48% of all diabetes‐related deaths occurring in people under 70 years. Approximately 460,000 deaths from kidney disease were linked to DM, and elevated blood glucose levels were responsible for nearly 20% of deaths from cardiovascular diseases [1]. In Ghana, a report indicated that DM is a leading medical cause of death, accounting for 2.58% of all deaths, with an age‐adjusted mortality rate of 36.81 per 100,000 population [2].

Metabolic syndrome (MetS) encompasses a collection of metabolic abnormalities indicated by several cardiovascular risk factors typically linked to central fat accumulation and insulin resistance [3]. As a consequence, MetS increases the risk of Type 2 diabetes mellitus (T2DM), cardiovascular diseases and stroke [3], thus representing a growing clinical and public health issue across the globe [4]. This phenomenon is partially linked to various factors, such as rising urban populations, excess energy consumption, growing rates of obesity and more sedentary lifestyle practices [4, 5]. Global estimates suggest that approximately one‐quarter of the worldwide population is affected by MetS [6], with estimates indicating a remarkable rise in MetS, mainly in developing nations [7]. Nonetheless, there are varying reports of the prevalence of MetS across Africa. For instance, Kalk and Jofe found that 47% of South Africans with T2DM have MetS [8]. In Nigeria, the rate of MetS among persons with T2DM was estimated to be 66% in Ibadan (7). In the Kumasi metropolis, 23.5% of people who reported at the outpatient department of five selected hospitals were found to have MetS [9].

Anaemia is the most common blood disorder in T2DM that may be linked to the kidney’s inability to produce sufficient amounts of erythropoietin [10]. The risk of developing anaemia in T2DM patients with kidney disease appears to arise sooner compared to patients with similar degrees of renal impairment from other causes [11, 12]. The mechanisms linking MetS to anaemia have been described in the literature. Anaemia and MetS are associated with systemic inflammation, decreased secretion of erythropoietin, injury to the renal tissue, symptomatic autonomic neuropathy, and related complications [13, 14]. Perhaps, the most important factor in the pathophysiology is the anti‐erythropoietin effect of cytokines in reducing the sensitivity of erythropoietin receptors and promoting erythrocyte apoptosis [15], leading to a reduction in Hb levels [16]. Additionally, nutritional complications arising from the interference of metformin in nutritional absorption [17], as well as renal complications of T2DM, are believed to accelerate the development of anaemia [18].

Despite the significance of anaemia and MetS in the pathogenesis of T2DM, the current literature is deficient regarding its burden. To address this gap, we designed this study to investigate the prevalence of anaemia and MetS comorbidity and associated factors among patients with T2DM in the Ho municipality. The findings of this study would be important for implementing interventional strategies to avert the onset of anaemia and related cardiovascular complications.

## 2. Materials and Methods

### 2.1. Study Design and Study Site

This hospital‐based cross‐sectional study was conducted from January 2022 to October 2022 at the Diabetic Clinic of Ho Municipal Hospital. The Ho Municipal Hospital is an agency of the Ghana Health Service located in Ho in the Ho Municipality of the Volta Region of Ghana. It is a secondary‐level health facility with a 120‐bed capacity. The hospital serves as a referral centre for health centres and community‐based health planning and services (CHPS) compounds (primary‐level health facilities) within the Ho municipality and those of the Republic of Togo. It provides services, including an outpatient department, an inpatient department, maternity services, an antenatal clinic, a diabetic clinic, a pharmacy, a herbal clinic, a laboratory and dietary counselling. At the time of this study, the diabetic clinic receives, on average, about 20 patients daily, with about 400 patients seen, including newly diagnosed patients and patients who have come for review.

### 2.2. Study Population and Sampling Technique

A convenient sampling method was used to recruit patients with T2DM for this study. The inclusion criteria were as follows: being 20 years or older, on hypoglycaemic medications and having hospital records available that validated their diagnosis. Patients with Type 1 diabetes mellitus (T1DM), below 20 years old and those having a history of chronic conditions, such as cardiovascular diseases, including heart failure, myocardial infarction and stroke, as well as other conditions, such as thyroid disease, acromegaly and cancers, were excluded from the study. Additionally, patients receiving hormonal therapy, lipid‐lowering medications or active treatments for infectious diseases, such as clinical malaria, as well as patients who were critically ill, those who had undergone blood transfusions during the last 3 months leading up to the time of the study and women who were menstruating or had their period in the week before the start of the study, were excluded from the study.

### 2.3. Sample Size Calculation

The Raosoft Online calculator was used to calculate the sample size for this study. A recommended minimum sample size of 193 was calculated from a population of 385 patients with T2DM, using a 95% confidence level, 5% margin of error and a response distribution of 50%.

### 2.4. Data Collection Methods and Instruments

The diabetes status of each participant was verified through medical records before the data collection process. In‐person interviews were carried out using a semistructured questionnaire to gather sociodemographic details, including age, sex, educational level, marital status, occupation and duration of T2DM. Data regarding participants’ lifestyles, such as physical activity, alcohol intake and dietary habits, were also collected. After an overnight fast (10–12 h), 6 mL of venous blood was collected from the antecubital fossa using a sterile, single‐use syringe and needle. About 2 mL of the blood was transferred into a serum gel separator and fluoride oxalate tubes, respectively. The blood samples were temporarily stored in Styrofoam containers without ice packs but shielded from sunlight. They were then centrifuged at 1000 rpm for 5 min at room temperature to obtain serum and plasma. The serum and plasma samples were used for biochemical analyses. The remaining 2 mL of whole blood was placed in an ethylenediaminetetraacetic acid (EDTA) tube to measure Hb levels. The biochemical analyses were performed on a fully automated Pro‐S Selectra Chemistry Analyzer (The Netherlands).

#### 2.4.1. Anthropometric and Blood Pressure Measurements

The anthropometric indices were assessed using methods outlined in the Anthropometric Standardization Reference Manual (ASRM) and the International Biological Program (IBP) [19]. The measurements were performed by two trained research assistants. Height was measured using a stadiometer with participants standing upright and feet together. Waist circumference (WC) was measured as the lowest circumference between the iliac crest and the rib cage. Hip circumference (HC) was measured as the circumference over the wider part of the buttock. The waist‐to‐height ratio was calculated as the WC divided by the height. BMI was measured on a bioelectrical impedance analysis (BIA) scale, which required the value of the measured height. The BIA device operates based on the resistance and reactance of bodily tissues when a small electrical current is passed through the body to measure fat content [20]. It uses eight electrodes arranged in a tetrapolar setup, where the participant stands barefoot on the scale, holds the display unit with both hands and extends their arms horizontally while maintaining an upright position.

#### 2.4.2. Biochemical Measurements

The plasma and serum aliquots were analysed for fasting blood glucose (FBG) and serum lipid profile. All biochemical measurements followed standardized laboratory procedures at the Clinical Chemistry Unit of the Ho Teaching Hospital Laboratory.

### 2.5. Definition of MetS Based on the Harmonized Equation

MetS was defined according to the proposed ‘harmonized’ criteria [21]. Participants must meet at least three of the following risk factors: increased WC (men: ≥ 94 cm, women: ≥ 80 cm), low HDL cholesterol (men: < 40 mg/dL [1 mmol/L], women: < 50 mg/dL [1.3 mmol/L]), hypertriglyceridemia ≥ 150 mg/dL (1.7 mmol/L), raised blood pressure (systolic BP ≥ 130 mmHg and diastolic BP ≥ 85 mmHg, or receiving treatment for hypertension) and elevated fasting plasma glucose (FPG ≥ 110 mg/d [5.6 mmol/L]) or DM.

### 2.6. Definition of Anaemia, Dietary Salt, Sugar, and Fat Intakes

The WHO definition for anaemia was adopted for this study (Hb levels less than 13 g/dL for males and less than 12 g/dL for females) [22]. Dietary salt intake was defined as self‐report of adding salt to a diet regularly. Dietary sugar intake was defined as self‐report of adding sugar to diet regularly. Dietary fat intake was defined as a self‐report of consuming red meat (used as a proxy for saturated fat) regularly.

### 2.7. Data Analysis

**FIGURE 1:**
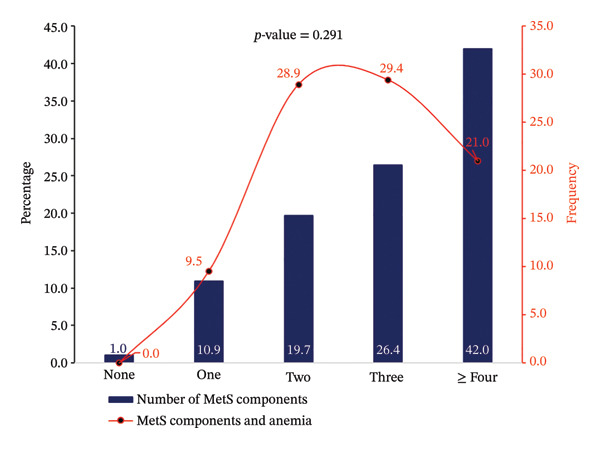
Variations in the frequency of anaemia in relation to the number of metabolic syndrome components.

Statistical analyses were performed using R software (Version 4.2.1; R Foundation for Statistical Computing, Vienna, Austria) with the packages tidyverse, broom, car, logistf, DescTools, gt, ggpattern and patchwork. Descriptive statistics were performed to summarize participant characteristics: continuous variables were presented as mean ± standard deviation (SD) as all assumed Gaussian distribution, and categorical variables as frequencies and percentages. As shown in Table 1, extreme outliers in continuous biomarkers were identified using Tukey’s rule (values below Q1 − 1.5 × IQR or above Q3 + 1.5 × IQR) and were excluded before the calculation of means, SDs and group comparisons; the same exclusion criteria were applied uniformly to both sexes to maintain comparability. Gender differences were assessed using the independent‐sample *t*‐test for continuous variables and the chi‐square test for categorical variables. The primary outcomes were (i) anaemia and (ii) coexistence of anaemia and MetS. For each outcome, three sequential multivariable logistic regression models were fitted. Model 1 included sociodemographic variables only (age, group, gender, marital status, education and occupation). Model 2 additionally adjusted for lifestyle factors (number of hours of work per day, exercise status, BMI category and current alcohol intake). Model 3 further added clinical variables (type of diabetic treatment and family history of diabetes). Results were presented as adjusted odds ratios (aORs) with 95% confidence intervals (CI). Model performance and stability were evaluated using the variance inflation factor (VIF) to assess multicollinearity, the Hosmer–Lemeshow goodness‐of‐fit test, Nagelkerke’s pseudo‐*R* ^2^ and the Akaike information criterion (AIC). The mean VIF was also calculated as an overall summary of collinearity. As shown in Figure 1, the association between the number of MetS components and the presence of anaemia was examined using a chi‐square test, with results displayed as a combined bar‐and‐line chart. All statistical tests were two‐sided, and a *p* value < 0.05 was considered statistically significant.

**TABLE 1:** Anthropometric, haemodynamic and biochemical characteristics of the study participants stratified by sex.

Parameters	Total	Males	Female	*p* values
Age (years)	50 ± 8.0	49 ± 9.0	51 ± 7.0	**0.0316**
Anthropometric				
Weight (kg)	75.7 ± 15.5	75.8 ± 14.9	75.3 ± 15.5	0.8224
Height (m)	1.63 ± 0.09	1.71 ± 0.08	1.59 ± 0.06	**< 0.0001**
BMI (kg/m^2^)	28.4 ± 5.8	25.8 ± 4.8	30 ± 5.8	**< 0.0001**
WC (cm)	93 ± 13.0	88 ± 16.0	95 ± 13.0	**0.0003**
HC (cm)	103 ± 13.0	98 ± 11.0	106 ± 14.0	**< 0.0001**
WHR	0.91 ± 0.05	0.92 ± 0.13	0.91 ± 0.11	**0.0175**
Haemodynamic				
SBP (mmHg)	127 ± 21.0	127 ± 22.0	126 ± 20.0	0.8428
DBP (mmHg)	81 ± 13.0	83 ± 13.0	80 ± 13.0	0.1830
HB (g/dL)	13.2 ± 1.7	14.3 ± 1.5	12.7 ± 1.3	**< 0.0001**
Biochemical				
FBG (mmol/L)	12.5 ± 5.3	13.4 ± 5.9	11.9 ± 4.9	0.0579
TC (mmol/L)	5.21 ± 1.18	4.67 ± 1.05	5.56 ± 1.12	**< 0.0001**
TrG (mmol/L)	1.57 ± 0.56	1.53 ± 0.58	1.69 ± 0.67	0.1028
HDL‐C (mmol/L)	1.41 ± 0.35	1.38 ± 0.36	1.43 ± 0.35	0.4135
LDL‐C (mmol/L)	3.01 ± 1.06	2.6 ± 1.07	3.27 ± 0.97	**< 0.0001**
VLDL‐C (mmol/L)	0.74 ± 0.3	0.71 ± 0.28	0.79 ± 0.33	0.0848
CRR	5.3 ± 1.4	5 ± 1.5	5.5 ± 1.2	**0.0100**

*Note:* Data are presented as mean ± standard deviation (SD). *p* values represent comparisons between males and females. A *p* value < 0.05 was considered statistically significant. Significant *p* values are in bold.

Abbreviations: BMI, body mass index; CRR, coronary risk ratio; DBP, diastolic blood pressure; FBG, fasting blood glucose; HB, haemoglobin; HC, hip circumference; HDL‐C, high‐density lipoprotein cholesterol; LDL‐C, low‐density lipoprotein cholesterol; SBP, systolic blood pressure; TC, total cholesterol; TrG, triglycerides; VLDL‐C, very low‐density lipoprotein cholesterol; WC, waist circumference; WHR, waist‐to‐hip ratio.

### 2.8. Ethical Issues

Approval for ethical considerations was secured from the Research and Ethical Review Committee at the University of Health and Allied Sciences, protocol number UHAS‐REC A.11 [16] 21‐22. The management of Ho Municipal Hospital also approved the study. The data collected for this study were maintained confidentially until the research was completed. The study was carried out according to the Helsinki Declaration.

## 3. Results

### 3.1. Sociodemographic and Behavioural Characteristics of the Study Participants

A total of 193 participants were recruited for this study. The majority were females, accounting for 119 (61.7%), and most were over the age of 50, with 110 (57.0%). Additionally, 132 participants (68.4%) were married. Most participants had attained basic education, represented by 102 (52.8%), and 142 (73.6%) were employed, while 53 (27.5%) worked more than 8 h a day, and 59 (30.6%) did not engage in any regular form of exercise. Dietary salt, sugar and fat intakes were 187 (96.9%), 104 (53.9%) and 160 (82.9%), respectively, among the study participants. Regarding current alcohol consumption, 40 (20.7%) reported drinking, and only 2 (1.0%) were smokers. The majority were on treatment with oral antihypoglycaemic medications only, totalling 131 (67.9%), and 129 (66.8%) reported having a family history of DM (Table 2).

**TABLE 2:** Sociodemographic and behavioural characteristics of the study participants.

Parameters	Frequency	Per cent
Total	**193**	**100.0**
Age (years)		
≤ 50	83	43.0
> 50	110	57.0
Gender		
Male	74	38.3
Female	119	61.7
Marital status		
Single	61	31.6
Married	132	68.4
Education level		
None	18	9.3
Basic	102	52.8
Secondary	38	19.7
Tertiary	35	18.1
Occupation		
Formal	41	21.2
Informal	10	5.2
None	142	73.6
Hours of work		
≤ 8 h	140	72.5
> 8 h	53	27.5
Exercise status		
Yes	134	69.4
No	59	30.6
Dietary salt intake		
No	6	3.1
Yes	187	96.9
Dietary sugar intake		
No	89	46.1
Yes	104	53.9
Dietary fat intake		
No	33	17.1
Yes	160	82.9
Alcohol intake (current)		
No	153	79.3
Yes	40	20.7
Smoking status (current)		
No	191	99.0
Yes	2	1.0
Type of treatment		
Oral only	131	67.9
Oral and insulin	62	32.1
Family history		
No	64	33.2
Yes	129	66.8

*Note:* Data were presented as frequency and proportion. Bold values represent the total frequency and corresponding percentage.

### 3.2. Anthropometric, Haemodynamic and Biochemical Characteristics of the Study Participants Stratified by Sex

The mean age of female participants was significantly higher than that of males (51 ± 7.0 vs. 49 ± 9.0 years; *p* = 0.0316). Except for weight, females demonstrated significantly higher mean anthropometric indices, including body mass index (BMI), WC and HC, whereas males had significantly greater height (*p* < 0.0001). The mean Hb levels were significantly higher in males compared to females (14.3 ± 1.5 vs. 12.7 ± 1.3 g/dL; *p* < 0.0001). Regarding biochemical parameters, total cholesterol (TC), low‐density lipoprotein cholesterol (LDL‐C) and coronary risk ratio (CRR) were significantly higher in females than males (*p* < 0.05) (Table 1).

### 3.3. Sex‐Stratified Prevalence of MetS and Its Individual Components

**FIGURE 2:**
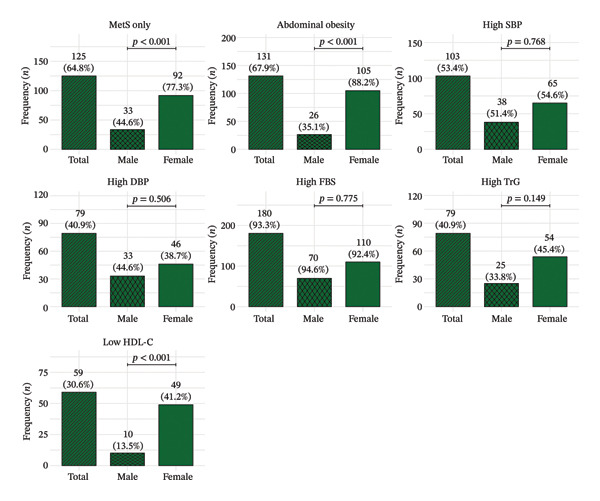
Prevalence of metabolic syndrome (MetS) and its individual components (abdominal obesity, high SBP, high DBP, high FBS, high TG and low HDL‐C) in the overall study population and stratified by sex. Bars represent frequencies (*n*) with corresponding percentages; *p* values indicate comparisons between males and females.

MetS was observed in 125 participants, representing 64.8% of the study population. The prevalence was significantly higher among females (92 [77.3%]) compared to males (33 [44.6%]; *p* < 0.001). Similarly, abdominal obesity was significantly more prevalent in females (105 [88.2%]) than males (26 [35.1%]; *p* < 0.001). In contrast, there were no significant sex differences in high SBP (females vs. males: 65 [54.6%] vs. 38 [51.4%]; *p* = 0.768), high DBP (46 [38.7%] vs. 33 [44.6%]; *p* = 0.506), high FBG (110 [92.4%] vs. 70 [94.6%]; *p* = 0.775) or high TrG (54 [45.4%] vs. 25 [33.8%]; *p* = 0.149). However, low HDL‐C levels were significantly more common among females (49 [41.2%]) compared to males (10 [13.5%]; *p* < 0.001) (Figure 2).

### 3.4. Prevalence of Anaemia Alone and Its Coexistence With MetS and Individual MetS Components Stratified by Sex

**FIGURE 3:**
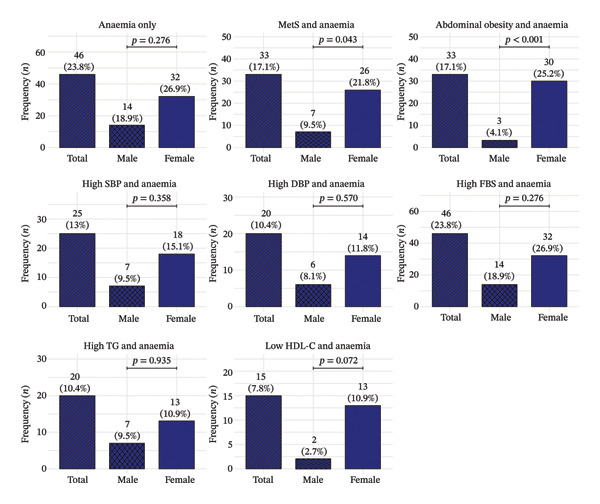
Prevalence of anaemia alone, coexistence of anaemia and metabolic syndrome (MetS) and anaemia in combination with individual MetS components (abdominal obesity, high SBP, high DBP, high FBS, high TG and low HDL‐C) in the overall study population and stratified by sex. Bars represent frequencies (*n*) with corresponding percentages; *p* values indicate comparisons between males and females.

The overall frequency of anaemia was 46 (23.8%) among the study participants. The coexistence of MetS and anaemia was observed in 33 (17.1%) participants, with a significantly higher prevalence among females (26 [21.8%]) compared to males (7 [9.5%]; *p* = 0.043). Abdominal obesity with anaemia was also significantly more common in females (30 [25.2%]) than males (3 [4.1%]; *p* < 0.001). Although females consistently demonstrated higher proportions across other MetS component–anaemia combinations, the differences were not statistically significant. Among the MetS components coexisting with anaemia, high FBG and anaemia constituted the most frequently observed combination (46 [23.8%]), followed by abdominal obesity and anaemia (33 [17.1%]) (Figure 3).

As shown in Figure 1, there were variations in the frequency of anaemia as the number of MetS components varied from no MetS component with 1.0% anaemia to one component with 9.5% anaemia, two components with 28.9% anaemia, three components with 29.4% anaemia and four or more components with 21.0% anaemia. However, the difference in the trend was statistically insignificant (*p* = 0.291).

### 3.5. Factors Associated With Anaemia Only and Its Coexistence With MetS

Table 3 presents the results of three sequential logistic regression models examining factors associated with anaemia only and the coexistence of MetS and anaemia. Female sex was significantly associated with higher odds of MetS–anaemia coexistence in Model 1 (OR = 3.51; 95% CI: 1.34–10.31). Current alcohol consumption was independently associated with substantially reduced odds of MetS–anaemia coexistence in Models 2 and 3 (OR = 0.10; 95% CI: 0.01–0.55 and 0.01–0.54, respectively). Model diagnostics indicated acceptable multicollinearity across models (mean VIF range: 1.31–1.41). The explained variance was modest, with Nagelkerke’s *R* ^2^ values ranging from 0.0715 to 0.1910 for the anaemia alone model and 0.0994 to 0.1837 for the MetS–anaemia model (Table 3).

**TABLE 3:** Multivariable logistic regression analysis of factors associated with anaemia alone and the coexistence of MetS and anaemia.

Parameters	Anaemia alone			Both MetS and anaemia		
	OR (95% CI)			OR (95% CI)		
	Model 1	Model 2	Model 3	Model 1	Model 2	Model 3
Age (years)						
≤ 50	1	1	1	1	1	1
> 50	0.34 (0.09–1.12)	0.35 (0.09–1.19)	0.34 (0.08–1.16)	1.42 (0.64–3.31)	1.47 (0.64–3.55)	1.52 (0.65–3.69)
Gender						
Male	1	1	1	1	1	1
Female	0.48 (0.13–1.82)	1.25 (0.26–6.26)	1.33 (0.27–6.97)	3.51 (1.34–10.31)^∗^	2.00 (0.69–6.26)	2.02 (0.69–6.39)
Marital status						
Single	1	1	1	1	1	1
Married	0.71 (0.22–2.51)	1.04 (0.30–3.99)	1.19 (0.32–4.93)	0.66 (0.29–1.52)	0.71 (0.30–1.72)	0.73 (0.30–1.81)
Education						
Below secondary	1	1	1	1	1	1
Secondary and above	0.88 (0.18–4.00)	0.96 (0.19–4.55)	1.02 (0.19–4.94)	1.44 (0.49–4.13)	1.18 (0.38–3.58)	1.20 (0.39–3.63)
Occupation						
Employed	1	1	1	1	1	1
Unemployed	1.35 (0.27–7.71)	1.00 (0.18–6.23)	0.93 (0.16–5.85)	0.46 (0.16–1.34)	0.43 (0.14–1.31)	0.39 (0.12–1.22)
Hours of work						
≤ 8 h		1	1		1	1
> 8 h		0.76 (0.15–2.91)	0.79 (0.16–3.12)		0.59 (0.20–1.54)	0.61 (0.20–1.61)
Exercise status						
Yes		1			1	1
No		0.99 (0.23–3.67)	1.10 (0.26–4.12)		0.64 (0.24–1.61)	0.62 (0.23–1.57)
BMI (kgm^−2^)						
Normal		1	1		1	1
Overweight		0.17 (0.02–0.87)^∗^	0.21 (0.03–1.15)		1.08 (0.35–3.48)	1.12 (0.34–3.74)
Obese		0.21 (0.04–0.95)^∗^	0.24 (0.04–1.13)		1.50 (0.52–4.57)	1.54 (0.51–5.00)
Alcohol intake current						
Yes		3.64 (0.84–16.04)	3.53 (0.79–15.98)		0.10 (0.01–0.55)^∗^	0.10 (0.01–0.54)^∗^
No		1	1		1	1
Diabetic treatment						
Oral only			1			1
Oral and insulin			2.52 (0.63–10.54)			1.39 (0.51–3.62)
Family history						
Yes			1.79 (0.43–8.48)			1.35 (0.51–3.86)
No			1			1
Model diagnostics						
Mean VIF	1.31	1.34	1.37	1.36	1.39	1.41
Nagelkerke^′^s *R* ^2^	0.0715	1.1669	0.1910	0.0994	0.1781	0.1837
AIC	101.7972	104.1610	106.2186	176.7197	176.4774	179.7561

*Note:* Values are presented as adjusted odds ratios (aORs) with 95% confidence intervals (CIs). Reference categories are indicated by OR = 1. Model 1 includes sociodemographic variables only; Model 2 additionally adjusts for lifestyle variables; and Model 3 further adjusts for clinical variables. A *p* value < 0.05 was considered statistically significant.

Abbreviations: AIC, Akaike information criterion; VIF, variance inflation factor.

^∗^Indicates statistically significant association.

## 4. Discussion

This study revealed a high prevalence of MetS (64.8%) among patients with T2DM in the Ho municipality, consistent with previous reports from Ghana (58%) [23] and Nigeria (66%) [24]. The predominance of MetS among females (77.3% vs. 44.6% in males) aligns with the significantly higher rates of abdominal obesity and low HDL‐C observed in women, underscoring the influence of gender‐specific adiposity patterns on metabolic risk.

The prevalence of anaemia was 23.8%, also comparable to the 24.4% reported previously in the Ho municipality [25] and within the range observed in other African diabetic populations [26]. The coexistence of anaemia and MetS (17.1%) was notably higher than the 3% reported among Chinese adults [27], likely reflecting the enriched risk profile of our T2DM population compared to the general population sample. The most frequent comorbidity combination was anaemia with elevated FBG (23.8%), consistent with the well‐documented relationship between hyperglycaemia and anaemia through mechanisms including oxidative stress, inflammation and impaired erythropoietin response [28].

Although the frequency of anaemia appeared to increase with accumulating MetS components, from 1.0% in participants with no MetS component to 29.4% in those with three, the trend did not reach statistical significance (*p* = 0.291). This finding contrasts with some studies [28] and may be attributable to the modest sample size and the relatively low anaemia prevalence. Alternatively, the lack of a clear dose–response relationship suggests that the presence of specific components, rather than the total count, may be more relevant to anaemia risk. This hypothesis is supported by the observation that hyperglycaemia, present in all participants, was the most common component co‐occurring with anaemia.

None of the examined sociodemographic, lifestyle or clinical variables demonstrated a statistically significant association with anaemia alone. The absence of significant predictors may reflect the multifactorial aetiology of anaemia in T2DM, which encompasses nutritional deficiencies, chronic kidney disease, inflammation and medication effects [16, 29]. Our inability to conduct iron studies, as well as measure vitamin B12 and inflammatory biomarkers, limited our capacity to delineate specific anaemia subtypes and their determinants.

Female sex was significantly associated with increased odds of anaemia–MetS comorbidity using the sociodemographic model (OR = 3.51; 95% CI: 1.34–10.31), indicating that females had approximately 351% higher odds of developing both conditions compared to males. However, this association attenuated and lost statistical significance after adjusting for lifestyle and clinical factors, indicating that the excess risk in women is largely explained by differences in adiposity, physical activity and other modifiable factors. This finding highlights the importance of targeting lifestyle interventions to high‐risk female patients.

The most striking finding was the strong, independent protective association between current alcohol consumption and anaemia–MetS comorbidity. In fully adjusted models, alcohol intake was associated with approximately 90% lower odds of developing the comorbidity (OR = 0.10; 95% CI: 0.01–0.54). This aligns with the well‐documented J‐shaped relationship between alcohol consumption and cardiovascular outcomes [30], and with evidence that moderate alcohol intake improves insulin sensitivity [31]. However, the mechanisms underlying the apparent protective effect against anaemia–MetS comorbidity remain speculative and may involve anti‐inflammatory effects, improved lipid profiles or enhanced erythropoiesis. Importantly, the small number of drinkers in our sample (*n* = 40) and the inability to quantify consumption levels warrant cautious interpretation and preclude causal inference. Residual confounding by unmeasured factors (e.g. socioeconomic status and dietary quality) cannot be excluded.

Overweight and obese BMI categories showed paradoxical protective associations with anaemia alone in Model 2 (OR = 0.17 and 0.21, respectively), but these effects were not sustained after full adjustment. This likely reflects confounding by other metabolic factors and the complex interplay between adiposity, inflammation and haematopoiesis [32].

The multivariable models demonstrated acceptable multicollinearity (mean VIF 1.31–1.41) but modest explanatory power (Nagelkerke’s *R* ^2^ 7%–19%), indicating that unmeasured factors contribute substantially to anaemia and its comorbidity with MetS.

Key limitations of this study include the cross‐sectional design (precluding causality), hospital‐based sampling (limiting generalizability), reliance on self‐reported lifestyle data (susceptible to recall bias) and absence of anaemia subtype biomarkers (iron, ferritin and B12). The small number of events for some outcomes may have limited statistical power to detect modest associations. Despite these limitations, this study provides perhaps the first assessment of anaemia–MetS comorbidity in a Ghanaian T2DM population, offering valuable baseline data for future research and highlighting the need for integrated screening and management strategies targeting both conditions.

## 5. Conclusion

This study demonstrates that anaemia and MetS comorbidity are highly prevalent among patients with T2DM in the Ho municipality, with nearly two‐thirds meeting criteria for MetS and approximately one‐quarter affected by anaemia. The coexistence of both conditions was observed in 17.1% of participants, which underscores the substantial burden of this comorbidity in clinical settings. The strong, independent protective association between alcohol consumption and reduced odds of anaemia–MetS comorbidity represents a novel finding that warrants cautious interpretation and further investigation. While this aligns with established cardioprotective effects of moderate alcohol intake, the small number of drinkers in our sample precludes definitive conclusions or clinical recommendations. The elevated risk observed in females was largely explained by differences in lifestyle and clinical factors, highlighting the importance of targeted interventions addressing modifiable risk factors in this subgroup. The absence of significant predictors for anaemia alone underscores the multifactorial aetiology of anaemia in T2DM and the need for comprehensive evaluation, including iron studies, inflammatory markers and renal function assessment. These findings support the integration of routine anaemia screening into diabetes management protocols and reinforce the importance of holistic, individualized care addressing both metabolic and haematological complications. Future prospective studies with larger samples, objective measures of alcohol consumption and comprehensive anaemia phenotyping are warranted to elucidate causal mechanisms and guide evidence‐based interventions.

## Author Contributions

Sylvester Yao Lokpo, David Avoyi, Eric Boateng, Samuel Ametepe, Kenneth Ablordey and Christian Obirikorang conceptualized and designed the experiments; carried out the experiments; provided reagents, logistics, analysis tools or data and wrote the paper.

Precious Kwablah Kwadzokpui analysed and interpreted the data and wrote the paper.

Esther Ngozi Adejumo, Michael Appiah, Francis Abeku Ussher, James Osei‐Yeboah and Abigail Ibrahim carried out the experiments, analysis tools or data and wrote the paper.

## Funding

No funding was received for this study.

## Disclosure

All authors of this paper have read and approved the final version submitted.

## Consent

The authors have nothing to report.

## Conflicts of Interest

The authors declare no conflicts of interest.

## Data Availability

The data that support the findings of this study are available from the corresponding author upon reasonable request.

## References

[1] Organization W. H. , Classification of Diabetes Mellitus, 2019.

[2] Expectancy W. L. , World Life Expectancy, 2016.

[3] Regufe V. M. , Pinto C. M. , and Perez P. M. , Metabolic Syndrome in Type 2 Diabetic Patients: A Review of Current Evidence, RISE Health Research Journal. (2020) 5, no. 6, 10.1097/j.pbj.0000000000000101. PMC7721212 33299950

[4] Khan A. , Global Epidemiology of Diabetes: The Role of Urbanization, Obesity, and Genetics, International Physiology. (2025) 13, no. 2, 70–81.

[5] Maboya M. W. , Razwiedani L. , Simbeni T. V. , Cele L. P. , and Mogale N. M. , Lifestyle Patterns and Dietary Habits of Patients Living With Type 2 Diabetes Mellitus Attending Primary Healthcare Facilities in Limpopo Province, South Africa, South African Journal of Clinical Nutrition. (2025) 38, no. 3, 155–162, 10.1080/16070658.2025.2539012.

[6] Hollman G. and Kristenson M. , The Prevalence of the Metabolic Syndrome and Its Risk Factors in a Middle-Aged Swedish Population—Mainly a Function of Overweight?, European Journal of Cardiovascular Nursing. (2008) 7, no. 1, 21–26, 10.1016/j.ejcnurse.2007.05.003, 2-s2.0-38949101433. 17586094

[7] Bowo-Ngandji A. , Kenmoe S. , Ebogo-Belobo J. T. et al., Prevalence of the Metabolic Syndrome in African Populations: A Systematic Review and Meta-Analysis, PLoS One. (2023) 18, no. 7, 10.1371/journal.pone.0289155. PMC10374159 37498832

[8] Kalk W. and Joffe B. , The Metabolic Syndrome, Insulin Resistance, and Its Surrogates in African and White Subjects With Type 2 Diabetes in South Africa, Metabolic Syndrome and Related Disorders. (2008) 6, no. 4, 247–255, 10.1089/met.2008.0003, 2-s2.0-57449109798. 19067527

[9] Fordah D. , Metabolic Syndrome and Associated Factors Among out Patients in Kumasi Metropolis, 2017, University of Ghana.

[10] Cawood T. , Buckley U. , Murray A. et al., Prevalence of Anaemia in Patients With Diabetes Mellitus, Irish Journal of Medical Science. (2006) 175, no. 2, 25–27, 10.1007/bf03167944, 2-s2.0-33745891169. 16872024

[11] El-Achkar T. M. , Ohmit S. E. , Mccullough P. A. et al., Higher Prevalence of Anemia With Diabetes Mellitus in Moderate Kidney Insufficiency: The Kidney Early Evaluation Program, Kidney International. (2005) 67, no. 4, 1483–1488, 10.1111/j.1523-1755.2005.00226.x, 2-s2.0-16244379523. 15780101

[12] Mahjoub A. , Patel E. , and Ali S. , Anemia in Diabetic Patients Without Underlying Nephropathy, 2016, A Retrospective Cohort Study American Society of Hematology.

[13] AlDallal S. M. and Jena N. , Prevalence of Anemia in Type 2 Diabetic Patients, Journal of Hematology. (2018) 7, no. 2, 57–61, 10.14740/jh411w. 32300413 PMC7155869

[14] Zhao L. , Zhang X. , Shen Y. , Fang X. , Wang Y. , and Wang F. , Obesity and Iron Deficiency: A Quantitative Meta‐Analysis, Obesity Reviews. (2015) 16, no. 12, 1081–1093, 10.1111/obr.12323, 2-s2.0-84947770747. 26395622

[15] Morceau F. , Dicato M. , and Diederich M. , Pro-Inflammatory Cytokine-Mediated Anemia: Regarding Molecular Mechanisms of Erythropoiesis, Mediators of Inflammation. (2009) 2009, no. 1, 405016–11, 10.1155/2009/405016, 2-s2.0-77952559404. 20204172 PMC2830572

[16] Shaheen E.-S. , Prevalence of Anemia in Patients With Type 2 Diabetes, Journal of Medicine in Scientific Research. (2019) 2, no. 2, 10.4103/jmisr.jmisr_29_19.

[17] Thomakos P. and Marinou K. , Long Term Metformin Use Association With Vitamin B12 Deficiency and Anemia, Journal of Diabetes, Metabolic Disorders and Control. (2017) 4, no. 1.

[18] Xie L. , Shao X. , Yu Y. et al., Anemia Is a Risk Factor for Rapid Egfr Decline in Type 2 Diabetes, Frontiers in Endocrinology. (2023) 14, 10.3389/fendo.2023.1052227. PMC9899800 36755908

[19] Lohman T. , Roche A. , and Martorell R. , Anthropometric Standardization Reference Manual, 1988, Human Kinetics.

[20] González-Correa C. H. , Body Composition by Bioelectrical Impedance Analysis, Bioimpedance in Biomedical Applications and Research, 2018, Springer, 219–241.

[21] Alberti K. G. , Eckel R. H. , Grundy S. M. et al., Harmonizing the Metabolic Syndrome: A Joint Interim Statement of the International Diabetes Federation Task Force on Epidemiology and Prevention; National Heart, Lung, and Blood Institute; American Heart Association; World Heart Federation; International Atherosclerosis Society; and International Association for the Study of Obesity, Circulation. (2009) 120, no. 16, 1640–1645, 10.1161/circulationaha.109.192644, 2-s2.0-70350245011. 19805654

[22] Geneva S. and Organization W. H. , Haemoglobin Concentrations for the Diagnosis of Anaemia and Assessment of Severity, Vitamin and Mineral Nutrition Information System Document Reference WHO. (2011) .

[23] Nsiah K. , Shang V. O. , Boateng K. A. , and Mensah F. , Prevalence of Metabolic Syndrome in Type 2 Diabetes Mellitus Patients, International Journal of Applied and Basic Medical Research. (2015) 5, no. 2, 133–138, 10.4103/2229-516x.157170. 26097823 PMC4456889

[24] Ipadeola A. and Adeleye J. , The Metabolic Syndrome and Accurate Cardiovascular Risk Prediction in Persons With Type 2 Diabetes Mellitus, Diabetes & Metabolic Syndrome: Clinical Research Reviews. (2016) 10, no. 1, 7–12, 10.1016/j.dsx.2015.08.011, 2-s2.0-84960086359. 26344942

[25] Lokpo S. Y. , Yarboye D. , Nkansah C. T. et al., Prevalence of Anaemia and Associated Factors Among Patients With Type 2 Diabetes Mellitus in the Ho Municipality in Ghana, Ghana Medical Journal. (2025) 59, no. 3, 151–158, 10.4314/gmj.v59i3.7. 41122257 PMC12536565

[26] Abate A. , Birhan W. , and Alemu A. , Association of Anemia and Renal Function Test Among Diabetes Mellitus Patients Attending Fenote Selam Hospital, West Gojam, Northwest Ethiopia: A Cross Sectional Study, BMC Blood Disorders. (2013) 13, no. 1, 10.1186/2052-1839-13-6, 2-s2.0-84962280961. PMC3816623 24499524

[27] Shi Z. , Hu X. , Yuan B. , Hu G. , Pan X. , and Holmboe-Ottesen G. , Coexistence of Anaemia and the Metabolic Syndrome in Adults in Jiangsu, China, Asia Pacific Journal of Clinical Nutrition. (2008) 17, no. 3, 505–513. 18818172

[28] Timerga A. , Haile K. , and Dessu S. , Anemia and Associated Factors Among Patients Admitted With Metabolic Syndromes at Worabe Comprehensive Specialized Hospital, Southern Ethiopia: A Cross-Sectional Study, PLoS One. (2022) 17, no. 4, 10.1371/journal.pone.0266089. PMC8979448 35377899

[29] Taderegew M. M. , Gebremariam T. , Tareke A. A. , and Woldeamanuel G. G. , Anemia and Its Associated Factors Among Type 2 Diabetes Mellitus Patients Attending Debre Berhan Referral Hospital, North-East Ethiopia: A Cross-Sectional Study, Journal of blood medicine. (2020) 11, 47–58, 10.2147/jbm.s243234. 32104127 PMC7023873

[30] Klatsky A. L. , Moderate Drinking and Reduced Risk of Heart Disease, Alcohol Research & Health. (1999) 23, no. 1, 15–23. 10890794 PMC6761693

[31] Avogaro A. , Watanabe R. M. , Dall’Arche A. , Vigili De Kreutzenberg S. , Tiengo A. , and Pacini G. , Acute Alcohol Consumption Improves Insulin Action Without Affecting Insulin Secretion in Type 2 Diabetic Subjects, Diabetes Care. (2004) 27, no. 6, 1369–1374, 10.2337/diacare.27.6.1369, 2-s2.0-2542448607. 15161790

[32] Muñoz-Ruiz M. A. , González-Zapata L. I. , Abril-Ulloa V. , and Gaitán-Charry D. A. , Metabolic Syndrome May Be Associated with a Lower Prevalence of Iron Deficiency in Ecuadorian Women of Reproductive Age, Journal of Nutritional Science. (2021) 10, 10.1017/jns.2020.55. PMC8057425 33889387

